# Mutant p53 protects ETS2 from non-canonical COP1/DET1 dependent degradation

**DOI:** 10.18632/oncotarget.7275

**Published:** 2016-02-09

**Authors:** Zunamys I. Carrero, Madhusudhan Kollareddy, Krishna M. Chauhan, Gopalakrishnan Ramakrishnan, Luis A. Martinez

**Affiliations:** ^1^ Department of Biochemistry, The University of Mississippi Medical Center, Jackson, MS 39216, USA; ^2^ Cancer Institute, University of Mississippi, Jackson, MS 39216, USA; ^3^ Department of Pathology and Cancer Center, Stony Brook University, Stony Brook, NY 11794, USA

**Keywords:** erythroblastosis virusE26 oncogene homologue 2 (ETS2), mutant p53, constitutive photomorphogenesis protein 1 (COP1), de-etiolated 1 (DET1), protein stability

## Abstract

Mutations in the tumor suppressor gene TP53 contribute to the development of approximately half of all human cancers. One mechanism by which mutant p53 (mtp53) acts is through interaction with other transcription factors, which can either enhance or repress the transcription of their target genes. Mtp53 preferentially interacts with the erythroblastosis virus E26 oncogene homologue 2 (ETS2), an ETS transcription factor, and increases its protein stability. To study the mechanism underlying ETS2 degradation, we knocked down ubiquitin ligases known to interact with ETS2. We observed that knockdown of the constitutive photomorphogenesis protein 1 (COP1) and its binding partner De-etiolated 1 (DET1) significantly increased ETS2 stability, and conversely, their ectopic expression led to increased ETS2 ubiquitination and degradation. Surprisingly, we observed that DET1 binds to ETS2 independently of COP1, and we demonstrated that mutation of multiple sites required for ETS2 degradation abrogated the interaction between DET1 and ETS2. Furthermore, we demonstrate that mtp53 prevents the COP1/DET1 complex from ubiquitinating ETS2 and thereby marking it for destruction. Mechanistically, we show that mtp53 destabilizes DET1 and also disrupts the DET1/ETS2 complex thereby preventing ETS2 degradation. Our study reveals a hitherto unknown function in which DET1 mediates the interaction with the substrates of its cognate ubiquitin ligase complex and provides an explanation for the ability of mtp53 to protect ETS2.

## INTRODUCTION

The majority of human cancers exhibit a loss of p53 function either as a result of mutations in the p53 gene (TP53) or due to dysfunctions in pathways that signal to p53 [1]. Mutated forms of p53 (mtp53) proteins not only exhibit a loss in wild-type functions, but also have dominant negative effect on the wild-type protein, rendering it inactive. Studies of p53's coding sequence have shown that more than 80% of its mutations occur in its DNA binding domain (DBD) [2]. The majority of TP53 mutations can be categorized into DNA contact and conformational mutations. DNA contact mutations are directly involved in DNA binding (e.g., R248W and R273H), and conformational mutations can cause local (e.g. R249S and G245S) or global (e.g., R175H and R282W) conformational distortions [3]. These changes provide multiple functions for mtp53 that can affect the genes that are transcribed by wild-type p53 and its interaction with other proteins (e.g. transcription factors). ChIP-on-chip and ChIP-seq analyses have shown that the predominant binding motif in mtp53 target genes is GGAAG, which also corresponds to the consensus-binding site for the erythroblastosis E26 transformation-specific (ETS) family of transcription factors [4]. The ETS family of transcription factors is found throughout the metazoan phyla, 28 genes of which are found in humans [5]. ETS proteins are sub-classified by the presence of different domains, which can be involved in protein-protein interactions (e.g. PNT domain) or transcriptional regulation (e.g., ETS domain) [6]. Previous studies have shown that mtp53 interacts with the ETS subfamily members, ETS erythroblastosis virus E26 oncogene homologue (ETS1) and ETS erythroblastosis virus E26 oncogene homologue 2 (ETS2) [4]. Mtp53 has been shown to preferentially bind to ETS2 and also to prevent its ubiquitin-dependent degradation. ETS2, an evolutionary conserved proto-oncogene and a downstream effector of the Ras/Raf/MAPK pathway, regulates the number of genes with potentially important functions in cancers such as: tumor environment, which includes growth factors, adhesion molecules, extracellular proteases and anti-apoptotic genes [7].

In this paper we wanted to characterize the mechanism by which mtp53 is stabilizing ETS2. We hypothesized that mtp53 stabilizes ETS2 by preventing its proteasomal degradation. By conducting a siRNA screening of most probable candidate ubiquitin ligases targeting ETS2, we identified the constitutive photomorphogenesis protein 1 (COP1) and the adaptor protein De-etiolated 1 (DET11) as negative regulators of ETS2 protein stability. COP1 is an E3 ubiquitin ligase which can directly or indirectly ubiquitinate its substrates with the aid of its RING finger domain. Most studies have shown that COP1's preferred mechanism of action consists of promoting substrate degradation through other E3 ligases [8]. It was shown that COP1 promoted the ubiquitination of transcription factors such as c-Jun and the ETS proteins ETS variant 1 (ETV1), ETS variant 4 (ETV4) and ETS variant 5 (ETV5) by recruiting them to the Damage-Specific DNA Binding Protein 1 (DDB1)–Cullin 4a (Cul4a) E3 ligase complex, an interaction mediated by the adaptor protein DET11 [8–10]. We determined that ETS2 is a substrate of the COP1/DET11 ubiquitin ligase complex.

There have been studies showing the regulation of ETS2 by ubiquitin ligases [11–13], but none describing the mechanism of its protection and stabilization by other proteins. Our data indicate that mtp53 reduces ubiquitination of ETS2 and protects it from proteasomal degradation by COP1/DET11. This leads to an accumulation of ETS2, which can cause an imbalance in ETS proteins that can lead to deregulation of important target genes involved in cancer progression. Understanding the dynamics between these two proteins will allow us to address possible target genes that may play important roles in the transition of normal cells to tumor cells. The possibility of disrupting this interaction or inhibiting the synergistic relationship between mtp53 and ETS2 may function as a new approach for possible therapeutic targets.

## RESULTS

### Ubiqutin ligase screening to identify regulator of ETS2 stability

**Figure 1:**
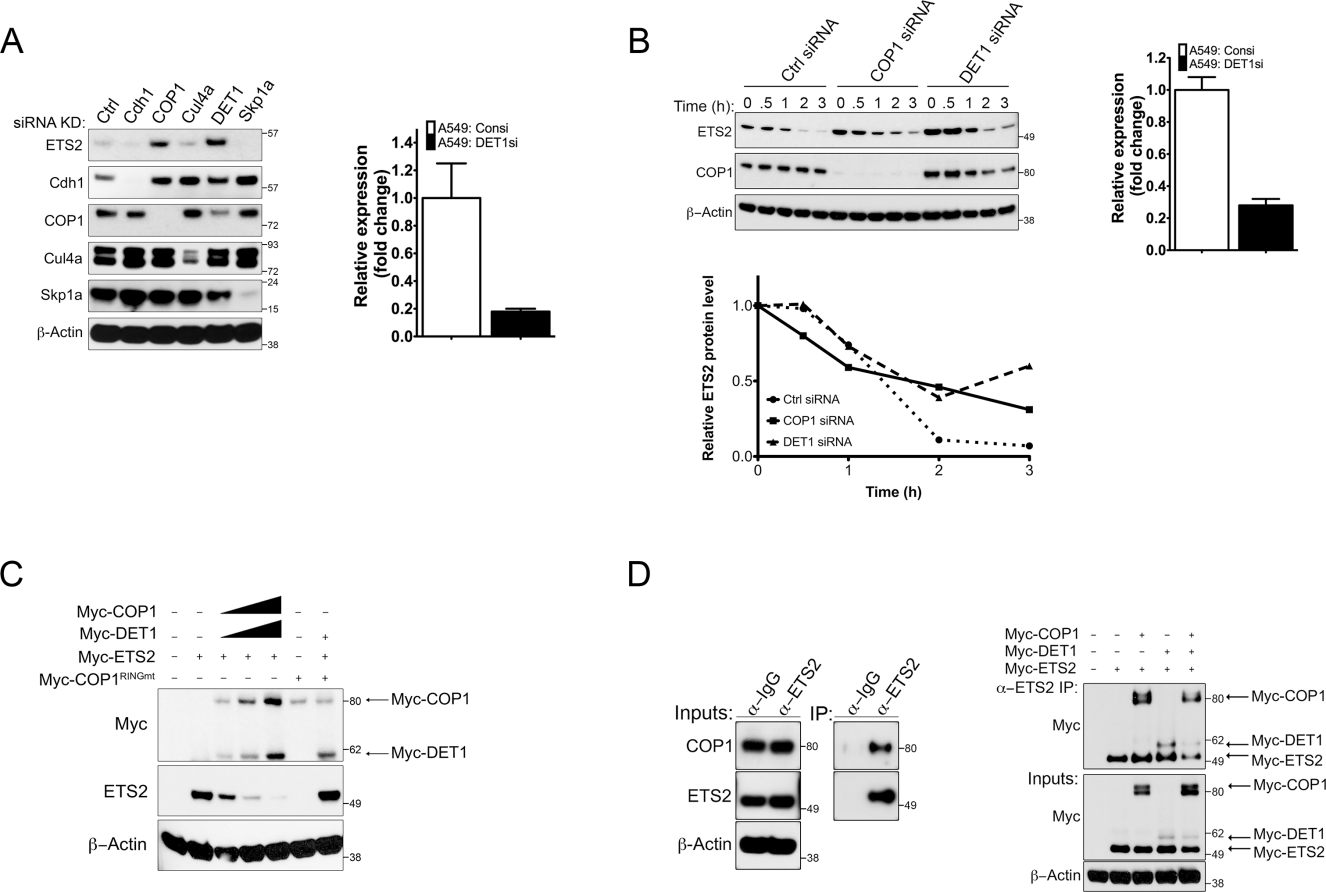
siRNA screening indicates COP1/DET1 are involved in ETS2 protein stability (**A**) ETS2 stability in the absence of candidate ubiquitin ligases. A549 (WTp53) cells were transfected with siRNAs targeting Cdhl, COP1, Cul4a, DET1 and Skpla for 48 h. Cells were lysed and processed for Western blotting. (A, right) Relative quantity expression of DET1 mRNA after DET1 siRNA knockdown for 48 h. (**B**) Cycloheximide (CHX) chase experiments for ETS2. (B, left) A549 cells were treated with control, COP1 and DET1 siRNA, for 48 h. Before harvesting, the cells were treated with MG-132 for l.5 h. After MG-132 treatment, the media was removed and replaced with fresh media containing l0 μg/mL of CHX. The samples were harvested at 0, 0.5, l, 2, 3 h followed by Western blotting. (B, bottom) Densitometry analysis with ImageJ for relative ETS2 protein levels normalized to the 0 h timepoint for each knockdown. (B, right) Relative quantity expression of DET1 mRNA after DET1 siRNA knockdown for 48 h. (**C**) Degradation of ETS2 by COP1/DET1. A549 cells were transfected with either wild-type COP1 or COP1 with the mutated RING residues Cl36A and Cl39A, DET1, and ETS2 for 24 h. Lysates were then harvested and processed for Western blotting. Solid triangle indicates increasing amounts of plasmid. (**D**) Co-immunoprecipitation of ETS2 with COP1 and DET1. (D, left) A549 cells were treated with MG-132 for 6 h followed by immunoprecipitation with anti-ETS2 (E-5) antibody. Immunoprecipitates were processed for Western blotting with anti-COP1 and anti-ETS2 antibody. (D, right) Lysates of PC3 (p53 null) cells transiently expressing COP1, DET1 and ETS2 for 24 h were immunoprecipitated with anti-ETS2 (C-20) antibodies. Prior to harvesting, the cells were treated with MG-132 for 6 h. Immunoprecipitates were processed for Western blotting with anti-Myc antibodies.

Previous studies have reported that ETS2 is a substrate of the APC/C and SCF ubiquitin ligase complexes. Mouse embryonic fibroblasts lacking Cdhl, the adaptor protein of the anaphase promoting complex (APC) E3 ubiquitin ligase, exhibit increased ETS2 protein stability [11]. Genetic and pharmacological analyses have also implicated Cul4a as a regulator of ETS2 stability. Additionally, Cul4a has been reported to interact with the adaptor protein of the SCF ubiquitin ligase complex, Skpla [12, 14]. Other studies have shown that the E3 ubiquitin ligase COP1 can bind to ETS family members and in the presence of its binding partner, DET1, promote their degradation [10, 15]. As an initial approach to understanding the control of ETS2 turnover, we examined the effect of knocking down selected ubiquitin ligases on ETS2 protein levels in A549 cells. Western blot analysis revealed that knockdown of either COP1 or its binding partner DET1 increased ETS2 protein levels (Figure 1A, lanes: 3, 5). We confirmed the knockdown of DET1 by real-time RT-PCR because we were unable to detect it using commercially available antibodies (Figure 1A, right).

### Proteasomal degradration of ETS2 by COP1/DET1

Next, we wanted to determine if the induction of ETS2 after siRNA knockdown of COP1 and DET1 was a result of increased ETS2 protein stability. We knocked down COP1 and DET1, and assessed the rate of ETS2 protein turnover. Transfected cells were treated with cycloheximide (CHX) and harvested at different time points for Western blot analysis (Figure 1B). In order to have the same amount of ETS2 at the first time point (0 or unt) measured under all conditions, we initially treated the cells with MG-132 to prevent its degradation and then added fresh media containing CHX. In control siRNA transfected cells, ETS2 protein levels almost completely disappeared after 1 hour of CHX treatment (Figure 1B, top left panel: lane 3, bottom left panel). With COP1 knockdown, ETS2 protein levels were higher at time points 0, 0.5, 1, and 2 hours (Figure 1B, top left panel: lanes 6–9, bottom left panel). The highest levels of ETS2 protein were observed in the DET1 knockdown samples. The knockdown of DET1 was verified by RT-PCR as above (Figure 1B, top right panel). These levels were approximately two times higher in time points 0, 0.5, and 1 hour than those seen in the control and COP1 knockdowns (Figure 1B, top left panel: lanes 11–13, bottom left panel). This suggests that ETS2 protein stability is controlled by an ubiquitin ligase complex containing COP1 and DET1. We did note that ETS2 was still degraded in the absence of COP1 and DET1, suggesting that there are other ubiquitin ligases that promote its degradation.

To confirm that COP1 and DET1 promote ETS2 degradation, we performed a degradation assay in which we co-transfected increasing amounts of Myc-COP1 COP1 and Myc-DET1DET1 with a fixed amount of Myc-ETS2 and assessed ETS2 levels by Western blot. We observed that co-transfection of COP1 and DET1 led to a decrease in ETS2 protein in a dose dependent manner (Figure 1C, lanes 2–5). Previously it has been reported that COP1 requires an intact RING domain to degrade its substrates including other ETS proteins [10, 16–18]. To address whether or not this was the case for ETS2, we generated a RING domain mutant [9, 10] and used it in the degradation assay. As with other ETS family members, ETS2 was not degraded by the COP1 RING mutant, confirming that a structurally intact RING domain is crucial for COP1 to promote ETS2 degradation (Figure 1C).

To establish that ETS2 is a direct substrate of COP1, we assessed if there was an interaction between the endogenous proteins. To capture the ETS2/COP1 complex, A549 cells were treated with MG-132 to prevent ETS2 destruction and then cell lysates were subjected to immunoprecipitation with an anti-ETS2 antibody. Western blot analysis showed that COP1 co-immunoprecipitated with ETS2 (Figure 1D, left).

COP1 and DET1 are found in a multisubunit ubiquitin ligase complex containing DDBl, Cul4a, and a putative undefined factor (referred to as X-box). In this context, DET1 is thought to associate with the DDBl/Cul4a ubiquitin ligase complex by interacting with the β-propellers in the DDBl protein's structure through a DET1 α-helical motif, H-Box [19]. The substrate specificity of this complex is imparted by the association of COP1 with specific binding motifs in the substrates. Hence, DET1 serves to assemble the multi-subunit ubiquitin ligase complex and COP1 functions as an adaptor subunit that recruits substrates for ubiquitination and subsequent degradation [9, 15].

Our siRNA data showed that DET1 knockdown also induced ETS2. Thus, we wanted to confirm that COP1, DET1 and ETS2 formed a complex. We performed co-immunoprecipitation experiments in PC3 cells, which lack COP1 [10, 20]. We transfected Myc-tagged versions of ETS2, COP1 and DET1 and then immunoprecipitated ETS2 to detect associated proteins. In agreement with the model in which COP1 binds to substrates, we detected an interaction between ETS2 and COP1 (Figure 1D, right: lane 3). Surprisingly, even though DET1's recognized function is to assemble the multi-subunit ubiquitin ligase complex, we detected an interaction between ETS2 and DET1 (Figure 1D, right: lane 4). Since these cells lack COP1, this data suggests that DET1 directly interacts with ETS2. Given that previous studies have reported that DET1 does not interact with substrates of this ubiquitin ligase complex, our data reveal a non-canonical activity of DET1 [9, 15].

### Domains in ETS2 required for COP1/DET1 degradation

**Figure 2:**
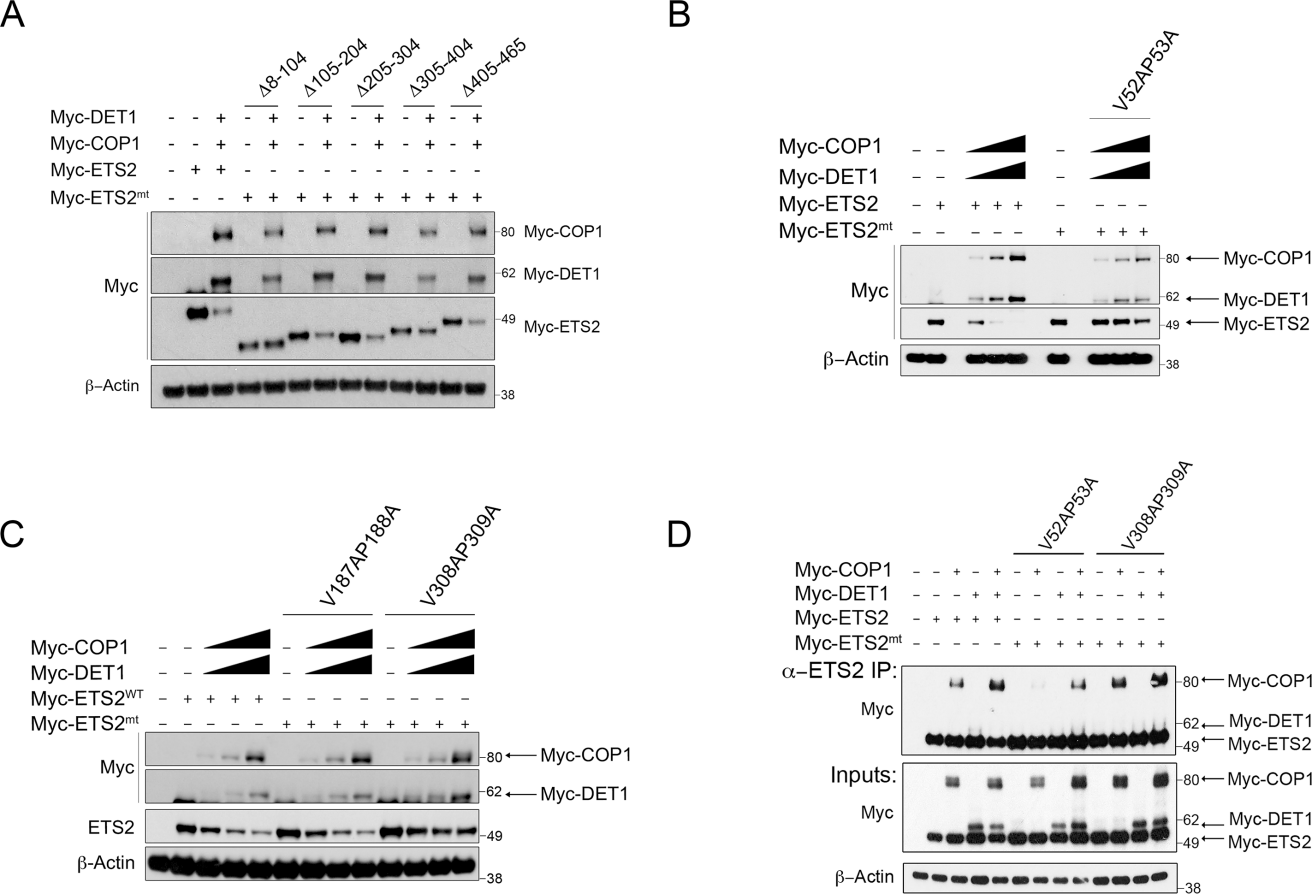
ETS2 amino acids V52, P53 and V308, P309 correspond to COP1 binding motifs required for degradation and interaction Degradation of ETS2 deletion and VP mutants by COP1/DET1. (**A**) A549 cells were transfected with either full-length ETS2 or with the mentioned ETS2 deletion constructs, COP1 and DET1, for 24 h. (**B**, **C**) A549 cells were transfected with either wild-type ETS2 or with the mentioned ETS2 VP constructs, COP1 and DET1 for 24 h. Lysates were then harvested and processed for Western blotting. (**D**) Lysates of PC3 cells transiently expressing COP1, DET1 and the mentioned ETS2 VP mutants were immunoprecipitated with anti-ETS2 (C-20) antibodies. The immunoprecipitates were processed for Western blotting with anti-Myc antibodies.

COP1 recognizes a degron motif that permits the interaction and subsequent degradation of its substrates. The amino acid sequence of ETS2 lacks a canonical COP1 degron motif; thus we took an unbiased approach to identify the residues required for its degradation. We tested the ability of COP1/DET1 to degrade Myc-tagged ETS2 deletion mutants that lack different portions of the protein (Δ8–l04, Δl05–204, Δ205–304, Δ305–404, Δ405–465). We found that the deletion mutant Δ8–l04 was protected from COP1/DET1 degradation (Figure 2A, lanes 4, 5). Similarly, the Δ305–404 deletion mutant, despite having lower expression levels, was relatively refractory to COP1/DET1 degradation (Figure 2A, lanes 10, 11). These data indicate that a sequence within this region is required for ETS2 degradation by the COP1/DET1 complex.

The degron motif V-P-E/D-X-G (X corresponds to a hydrophobic residue) is required for the recognition of substrates by COP1 [21]. As the Δ8–l04 and Δ305–404 deletion mutants were not degraded by COP1/DET1, we searched within the deleted regions for he conserved VP residues. We identified two putative partial COP1 degron motifs, one at amino acids 52 and 53 (V52,P53) and another in amino acids 308 and 309 (V308,P309). As this did not constitute a complete COP1 degron motif, we opted to search the rest of the ETS2 sequence for a similar motif to use as a control, identifying residues in amino acids l87 and l88 (Vl87,Pl88).

We mutated these putative COP1 degron sites to alanines (V52A,P53A, Vl87A,Pl88A, and V308A,P309A) and tested them in the degradation assay. The V52A,P53A mutant was not degraded by COP1/DET1 (Figure 2B, lanes 6–9). The V308A,P309A was partially resistant to degradation (Figure 2C, lanes 10–13) but the Vl87A,Pl88A was not (Figure 2C, lanes 6–9). These data agree with our results above and indicate that a degron motif is harbored within amino acids 8–l04 and 305–404 of ETS2.

To test if the ETS2 VP mutants were protected from degradation because they were not recognized by COP1, we assessed their ability to interact with COP1 and DET1 in the PC3 cells. The interaction between the ETS2 V52A,P53A mutant and COP1 was severely compromised (Figure 2C, lane 7) but the V308A,P309A mutant bound as well as wild-type ETS2 (Figure 2C, lanes: 3,11). Strikingly, mutation of either of these VP sites virtually eliminated the interaction with DET1 (Figure 2D, lanes: 8, 12). The fact that the mutation of these sites protects ETS2 from COP1/DET1 mediated degradation suggests that the interaction with DET1 is required for ETS2 destruction. Therefore, DET1 plays a non-canonical role in the degradation of ETS2 in that it not only serves by forming a bridge between the ubiquitin ligase complex and COP1, but also by serving as a substrate-binding adaptor.

### COP1 participates in CDK10 destruction of ETS2

**Figure 3:**
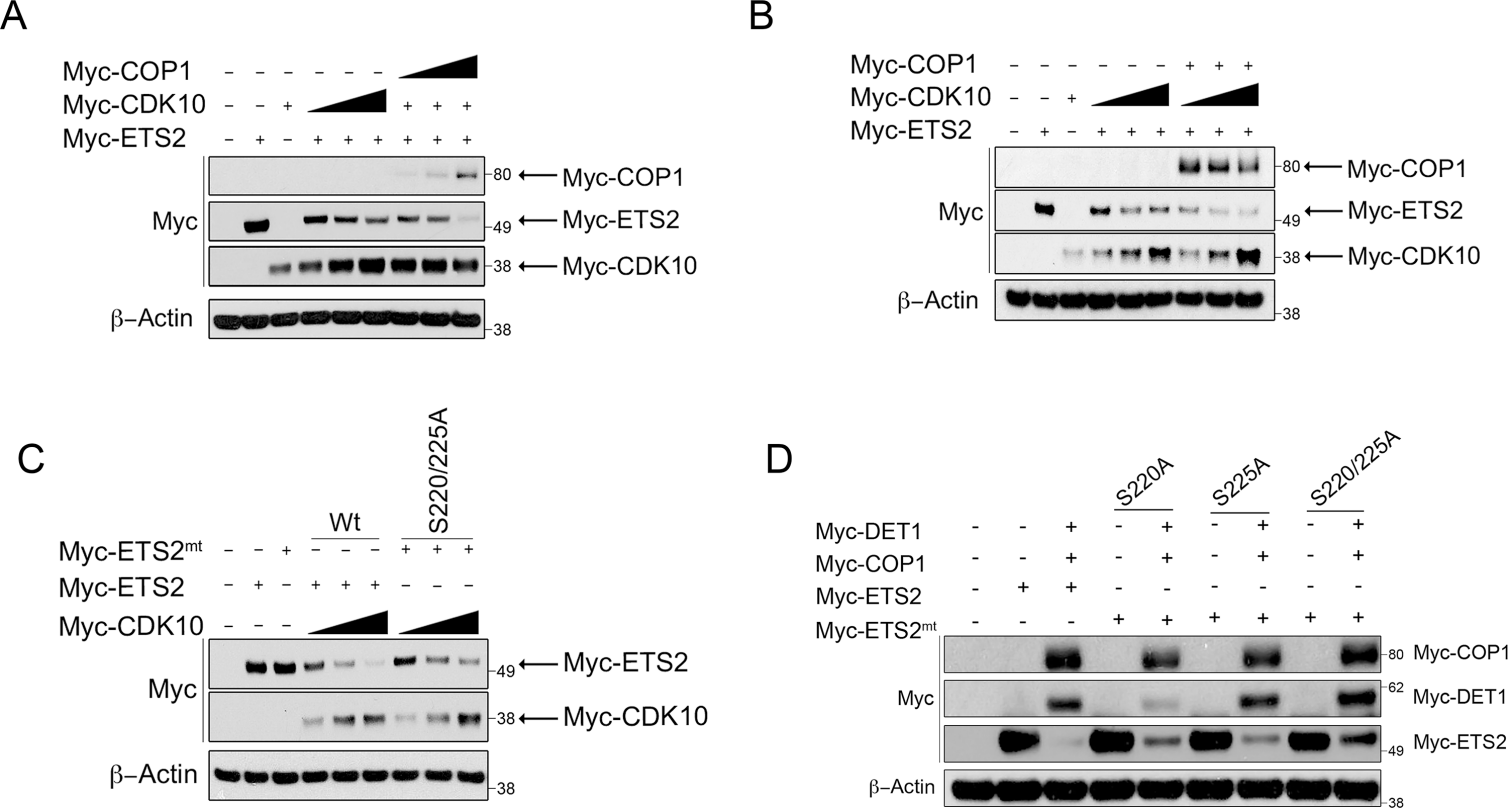
COP1/DET1 aid in the CDK10 mediated degradation of ETS2 Degradation of ETS2 wild-type and alanine mutants by COP1/DET1 and CDK10. (**A**) PC3 cells were transfected with wild-type ETS2, CDK10, and a set amount of CDK10 with increasing amounts of COP1 for 24 h. (**B**) PC3 cells were transfected withwild-type ETS2, CDK10, and a set amount of COP1 with increasing amounts of CDK10. (**C**) A549 cells were transfected with either wild-type ETS2 or with the mentioned ETS2 alanine mutants and CDK10 for 24 h. (**D**) A549 cells were transfected with either wild-type ETS2 or with the mentioned ETS2 alanine mutants, COP1 and DET1, for 24 h. (A, B, C, D) Lysates were then harvested and processed for Western blotting. Solid triangle indicates increasing amounts of plasmid.

Previous studies have reported that the cyclin dependent kinase l0 (CDK10) negatively regulates ETS2's transactivation activity and that it can phosphorylate ETS2 and promote its ubiquitin-dependent degradation [13, 22–24]. However, the ubiquitin ligase that cooperates with CDK10 to degrade ETS2 is not known. We confirmed that co-expression of CDK10 and ETS2 leads to degradation of the latter (Figure 3C, lanes: 4–6). In contrast to what was observed in COP1 expressing cells, increasing amounts of CDK10 in COP1 null PC3 cells yielded only a modest decrease in ETS2; moreover, that decrease plateaued and failed to completely eliminate ETS2 (Figure 3A, lanes: 4–6). To determine if CDK10 requires COP1/DET1 to promote ETS2 degradation, we determined if co-transfection of COP1 facilitated CDK10 degradation of ETS2. We found that co-transfection of COP1 with CDK10 and ETS2 resulted in complete elimination of ETS2, suggesting that COP1 is required for CDK10 to fully promote ETS2 degradation (Figure 3A, 3B, lanes: 7–9).

Having established that COP1 mediates the destruction of ETS2, we wanted to determine any existing interplay between the CDK10 phosphorylation sites in ETS2 and the ability of COP1/DET1 to mediate its degradation. CDK10 has been shown to phosphorylate multiple sites in ETS2, but most notably, serines 220 and 225 appear to be important to ETS2 degradation [13]. Co-transfection of CDK10 with an ETS2 mutant in which we changed these serines to alanines (S220A,S225A) revealed that mutation of these amino acids provides some protection from degradation (Figure 3C, lanes: 7–9). Likewise, mutation of serine 220 and 225, either alone or combined, provided protection of ETS2 from COP1 degradation (Figure 3D, lanes: 5, 7, 9). As CDK10 reportedly phosphorylates other sites (serines 246, 248, 255 and 3l9) in ETS2 [13], we tested whether or not they regulated ETS2 destruction.

**Figure 4:**
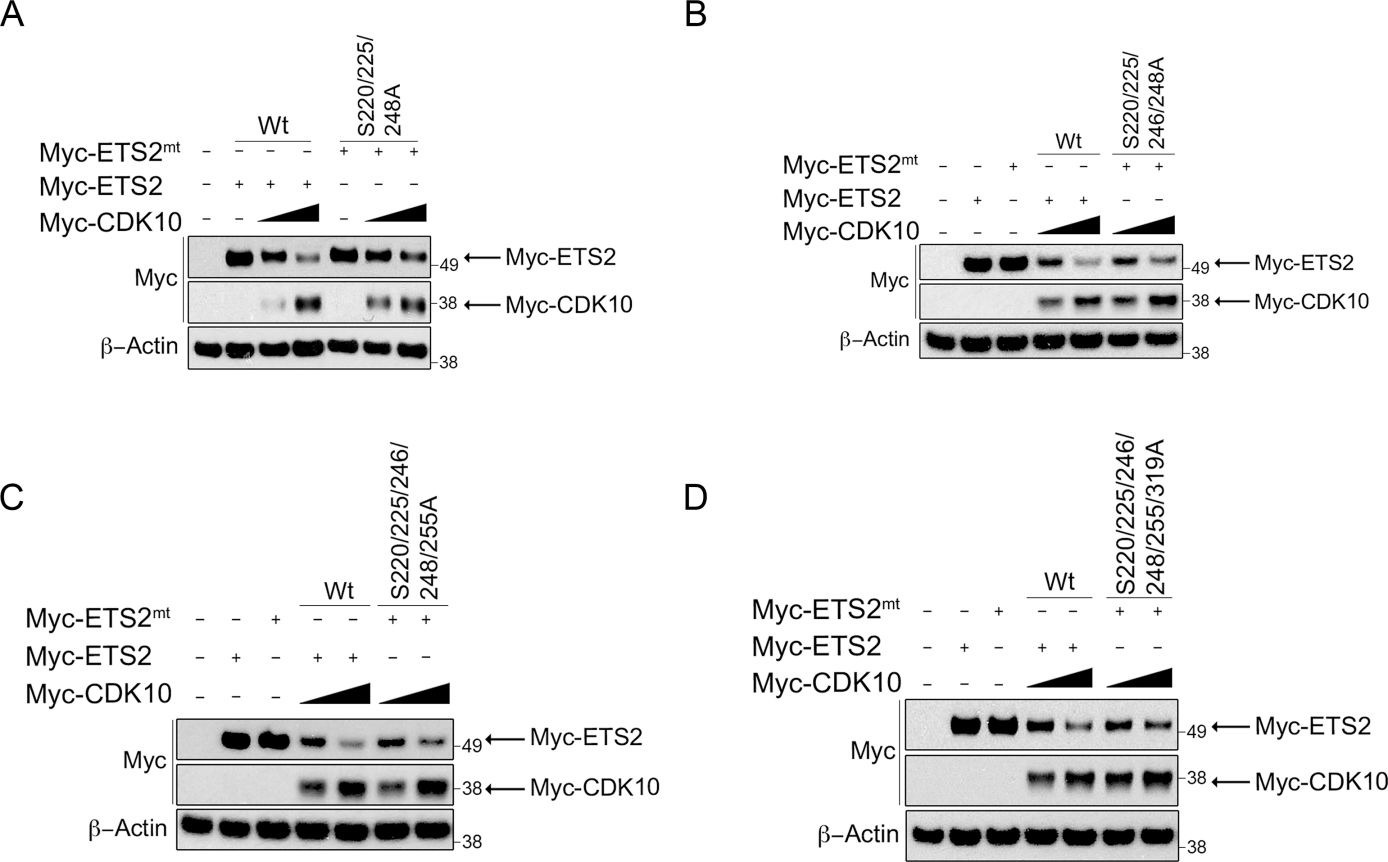
ETS2 alanine mutants for CDK10 phosphorylation sites are less susceptible to CDK10 mediated degradation (**A**, **B**, **C**, **D**) CDK10 mediated degradation of ETS2 alaninemutants.A549 cells were transfected with either wild-type ETS2 or withthementioned ETS2 alanine mutants and CDK10 for 24 h. Lysates were then harvested and processed for Western blotting. Solid triangle indicates increasing amounts ofplasmid.

Individual mutation of these amino acids failed to consistently yield protection from CDK10 degradation (data not shown). Our observation that mutation of serine 220 or 225 alone provided a small degree of protection for ETS2 led us to speculate that phosphorylation of a certain combination of these residues cooperatively regulates ETS2 stability. Therefore, we mutated different combinations and again assessed whether or not they were protected from degradation (Figure 4A–4D). Of the different combinations, we noted that mutation of serines 220, 225 and 248 provided the highest protection in the degradation assay (Figure 4A, lanes: 6, 7) compared to mutation combinations of serines 220, 225, 246, 248 (Figure 4B, lanes: 6, 7) and 220, 225, 246, 248, 255 (Figure 4C, lanes: 6, 7). The mutant affording the second highest level of protection from degradation was the ETS2 phosphorylation mutant, in which all the putative CDK10 phosphorylation sites described were mutated to alanines (Figure 4D, lanes: 6, 7). Since mutation of these sites conferred protection from degradation, our data suggest their phosphorylation by CDK10 (S220, S225, S248) likely marks ETS2 for COP1/DET1 mediated degradation.

**Figure 5:**
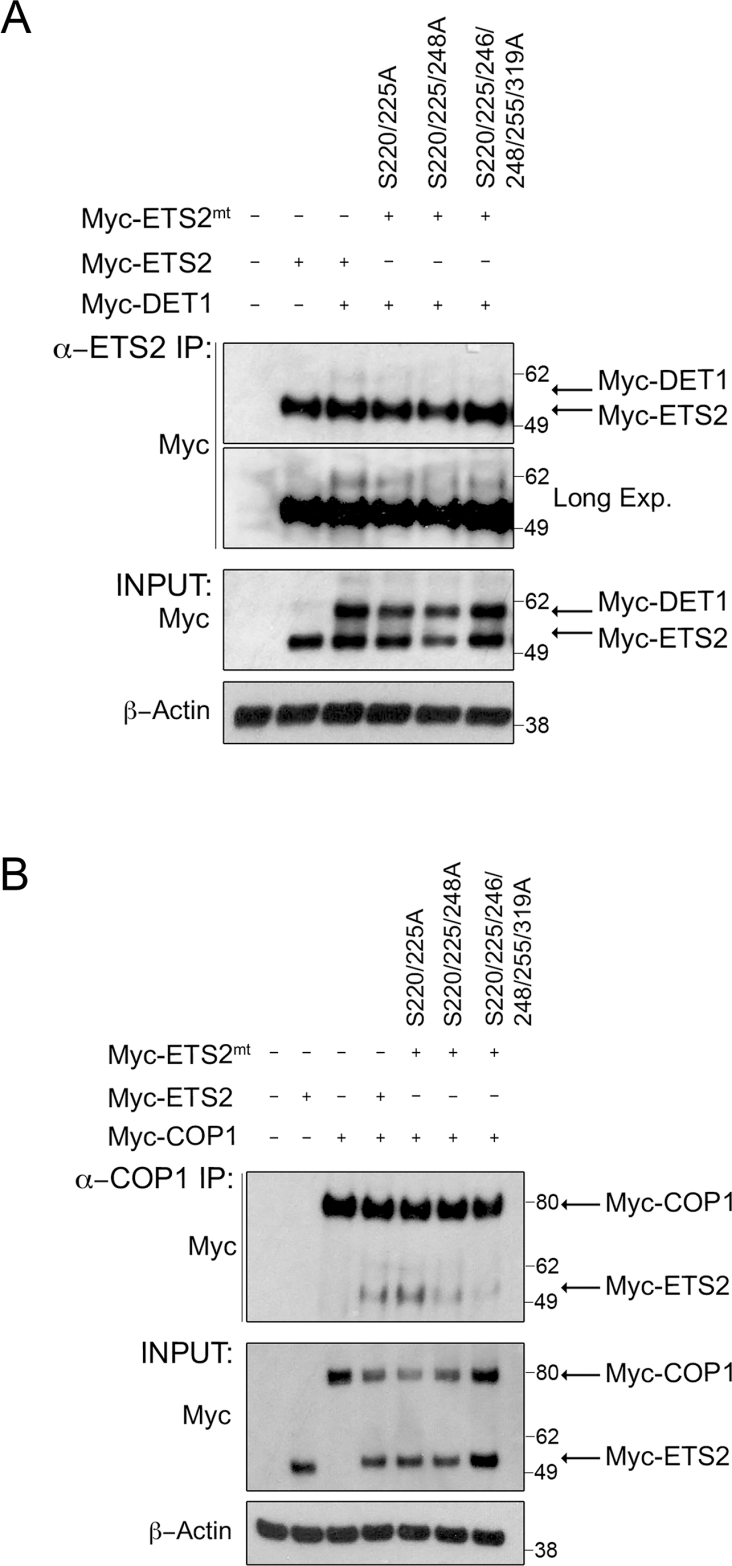
Alanine mutations of CDK10 serine phosphorylation sites in ETS2 disrupt DET1 binding and reduces COP1 interaction Co-immunoprecipitation of ETS2 wild-type or the mentioned alanine mutants with DET1 (**A**) and COP1 (**B**). PC3 cells were transfected with either wild-type ETS2 or the mentioned alanine mutants and DET1 (A) or COP1 (B) for 24 h. Samples were treated with MG-132 for 6 h prior to harvesting. Lysates were then immunoprecipitated with anti-ETS2 (C-20) antibodies (A) or anti-COP1 antibodies (B) and then processed for Western blotting.

We demonstrated above that DET1 interacts with ETS2 independently of COP1 and that mutation of the minimal consensus sequence of COP1 degrons eliminates the ETS2/DET1 interaction and prevents ETS2 degradation. These data suggest that the interaction between ETS2 and DET1 is critical for ETS2 degradation; thus we next sought to determine if ETS2 could be protected from degradation by preventing the interaction between serine triple mutant (S220, S225, S248) and DET1. To test this possibility, we compared the interaction between several ETS2 phosphorylation mutants and DET1. Mutation of serines 220 and 225 resulted in a pronounced decrease in the ETS2 and DET1 interaction (Figure 5A, lane 4). Moreover, mutation of serines 220, 225 and 248 further reduced this interaction (Figure 5A, lane 5). Mutation of additional residues (serines 246, 255 and 3l9) did not prevent the association of ETS2 with DET1 (Figure 5A, lane 6). We proceeded to also assess the interaction between the ETS2 phosphomutants and COP1. COP1 was not affected by ETS2 mutations in serines 220 and 225 (Figure 5B, lane 5). However, mutants (serines 220, 225, 248) (Figure 5B, lane 6) and (serines 220, 225, 246, 248, 255, 3l9) (Figure 5B, lane 7) showed a decrease in interaction. Our data therefore suggest that phosphorylation of serines 220, 225 and 248 in part serves as the signal codifying ETS2 for destruction.

### Mutant p53 protects ETS2 from COP1/DET1 degradation

**Figure 6:**
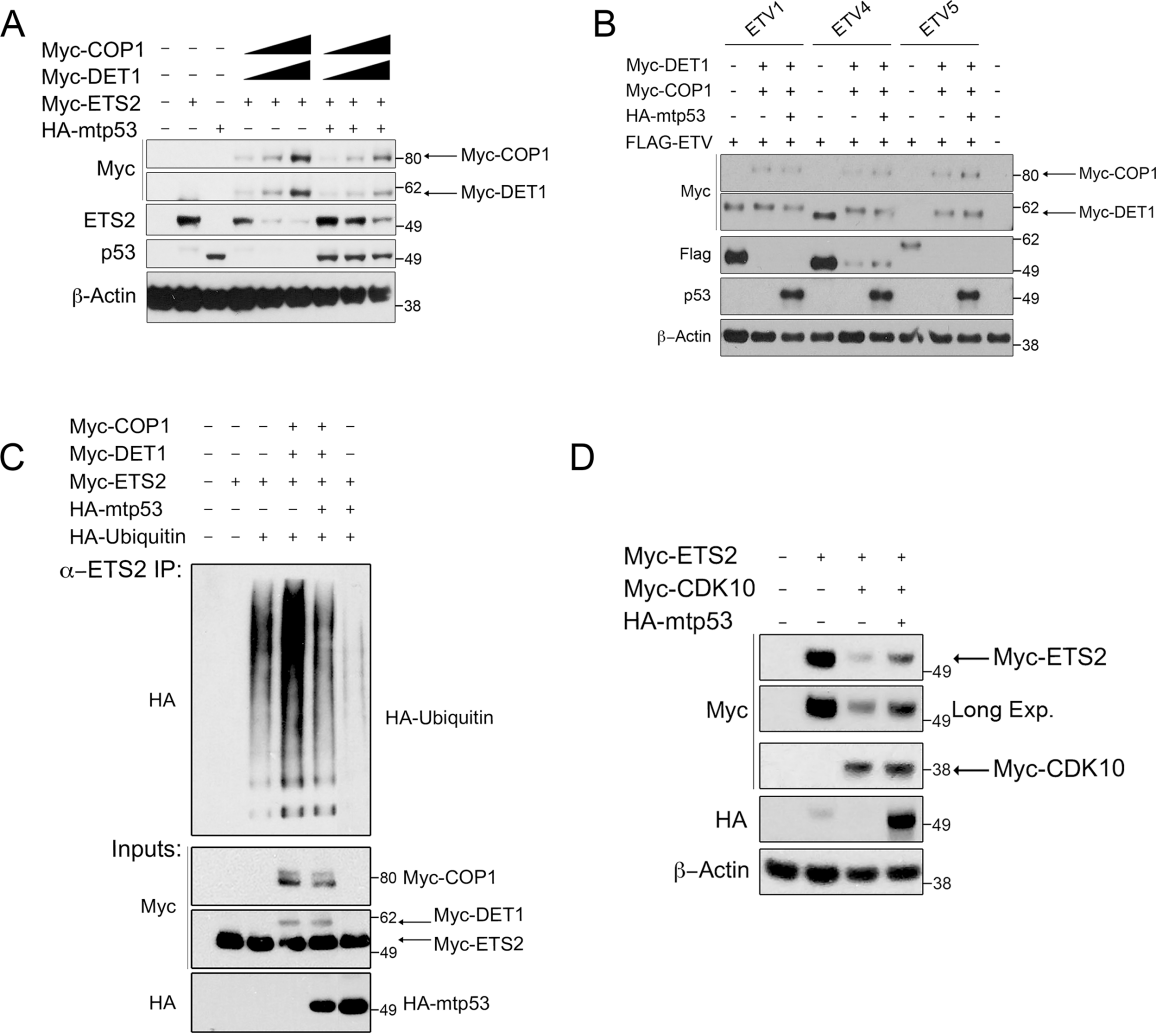
Mtp53 decreases ubiquitination and protects ETS2, and not other ETS proteins, from COP1 and DET1 degradation Degradation and ubiquitination assay of ETS2. (**A**) A549 cells were co-transfected with ETS2, COP1 and DET1 with or without mtp53 R248W. Cells were harvested after 24 h, followed by Western blotting. Solid triangle indicates increasing amounts of plasmid. (**B**) A549 cells were co-transfected with ETVl, ETV4, or ETV5, and each with COP1 and DET1, with and without mtp53 R248W. Cells were harvested after 24 h, followed by Western blotting. (**C**) A549 cells were transfected for 24 h with ETS2, poly ubiquitin, COP1, DET1, and mtp53 R248W. Prior to harvesting, cells were treated with MG-132 for 6 h. Cells were harvested under denaturing conditions by using boiling l% SDS buffer, which was then diluted to RIPA buffer. Lysates were then immunoprecipitated with anti-ETS2 (C-20) antibodies. Immunoprecipitates were processed for Western blotting with anti-HA antibodies. (**D**) Mtp53 protects ETS2 for CDK10 mediated degradation. CDK10 mediated degradation assay of ETS2. A549 cells were co-transfected with ETS2 and CDK10 with or without mtp53 R248W. Cells were harvested after 24 h, followed by Western blotting.

Previously we reported that mtp53 interacts with ETS2 and protects it from uibiquitin-mediated degradation [4]. Importantly, WTp53 interacts poorly with ETS2 and it does not prevent degradation of the latter [4]. Using a series of deletion mutants, we showed that mtp53 binds to ETS2 in a region encompassing amino acids 225–264. Given the overlap between the mtp53 binding region and the CDK10 phosphorylation sites in ETS2, we speculated that mtp53 stabilizes ETS2 by masking these sites from being recognized by DET1. By assuming this to be so, we further predicted that mtp53 should protect ETS2 from COP1/DET1 mediated degradation. Co-transfection of ETS2 with increasing amounts of COP1/DET1 led to ETS2 destruction, but importantly, when we included mtp53 in the degradation assay we observed that mtp53 potently prevented ETS2 degradation (Figure 6A, lanes: 7–9 and Supplementary Figure 1, lanes: 7–9).

Although we speculated that mtp53 protects ETS2 by masking the region bound by the COP1/DET1 complex, an alternative mechanism by which mtp53 protects ETS2 could be by inactivating COP1 ubiquitin ligase activity. To address this possibility, we performed degradation assays with the other ETS family members (ETVl, ETV4, and ETV5), which are known substrates of this ubiquitin ligase complex: mtp53 failed to protect these other ETS family members, suggesting that it only protects ETS2 (Figure 6B). The continued degrading of ETVl, ETV4 and ETV5 by COP1/DET1 indicates that mtp53 does not inhibit the ubiquitin ligase activity of this complex.

To further substantiate that mtp53 prevents the ubiquitin-mediated destruction of ETS2, we performed an ubiquitination assay by transfecting all components used in the degradation assay, including HA-tagged ubiquitin, in order to observe the extent of ubiquitination. For this experiment, ETS2 was immunoprecipitated under denaturing conditions to prevent the co-precipitation of other ubiquitinated proteins; this done, we detected ubiquitination with an antibody against the HA-tag. Transfection of ETS2 with HA-ubiquitin revealed that ETS2 is actively ubiquitinated (Figure 6C, lane 3). When we also included COP1/DET1 in the transfection, we observed markedly increased ubiquitination (Figure 6C, lane 4). In agreement with the ability of mtp53 to protect ETS2 from degradation, we further observed that the inclusion of mtp53 in the transfection largely reduced the extent of ubiquitination (Figure 6C, lane 5). Of note, co-transfection of mtp53 with ETS2 and HA-ubiquitin virtually eliminated the basal ubiquitination level (Figure 6C, lane 6). Taken together, our data indicate that mtp53 specifically prevents the COP1/DET1 mediated ubiquitination and degradation of ETS2.

**Figure 7:**
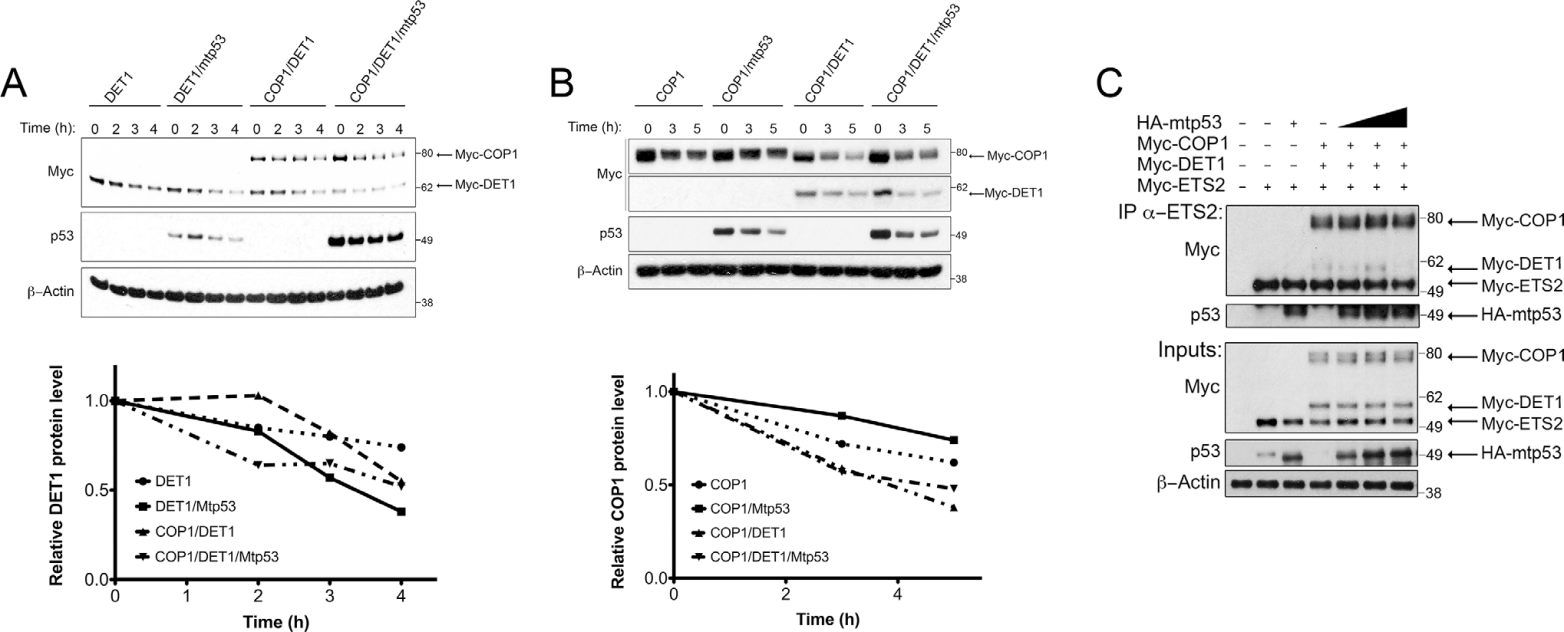
Mtp53 destabilizes DET1 and blocks the interaction between DET1 and ETS2 (**A**, **B**, top) A549 cells were transfected with the indicated plasmids and protein turnover was assessed after blocking new protein synthesis with CHX. (A, bottom) Densitometry analysis for relative DET1 or COP1 (B, bottom) protein levels normalized to the 0 h timepoint for each tranfection. (**C**) Co-immunoprecipitation of ETS2 with COP1, DET1, with or without mtp53. PC3 cells were transfected with ETS2, COP1, DET1, and mtp53 R248W for 24 h. Samples were treated with MG-132 for 6 h prior to harvesting. Lysates were then immunoprecipitated with anti-ETS2 (C-20) antibodies and then processed for Western blotting. Solid triangle indicates increasing amounts of plasmid.

Consistent with the ability of mtp53 to protect ETS2, we observed that mtp53 partially rescued ETS2 from CDK10 (Figure 6D, lane 4). We noticed that in the presence of mtp53, both COP1 and DET1 were expressed at lower levels. To determine whether or not mtp53 affected the stability of these proteins, we co-transfected them and examined their half-life in the presence of mtp53. Densitometry analysis revealed that just after 2 hours, DET1 had a markedly higher turnover in the presence of mtp53, suggesting that the protein was less stable (Figure 7A, top lanes: 5–8 and bottom). In a separate experiment, Western blot analysis revealed that COP1 protein turnover was not affected to the same magnitude as was DET1 by the presence of mtp53 (Figure 7B, top lanes: 4–6 and bottom). The results of these experiments suggested that mtp53 protects ETS2 from degradation by destabilizing DET1. If so, we would expect to find less DET1 bound to ETS2 in the presence of mtp53.

To directly assess this possibility, we co-transfected the COP1 null PC3 cells [20] with different combinations of COP1, DET1, ETS2 and mtp53 to assess their interactions. As before, we could detect the interaction between COP1/DET1 and ETS2 by immunoprecipitation. In contrast, the inclusion of increasing amounts of mtp53 in this transfection reduced the amount of DET1 that co-precipitates with ETS2 (Figure 7C, lanes: 5–7). Nevertheless, the interaction between ETS2 and COP1 was only minimally affected.

Our early results in the present work suggested that an association of DET1 with ETS2 is required for ETS2 degradation. The role of mtp53 in reducing DET1 levels suggests that mtp53 protects ETS2 from degradation by controlling the abundance of DET1, a critical component of the ubiquitin ligase complex. In addition, the fact that mtp53 and DET1 appear to bind to the same region in ETS2 suggests that the proteins may compete for binding to these sites. Such competition, in tandem with the reduction of DET1 levels, would allow mtp53 to protect ETS2 from destruction by disrupting the interaction between DET1 and ETS2.

**Figure 8:**
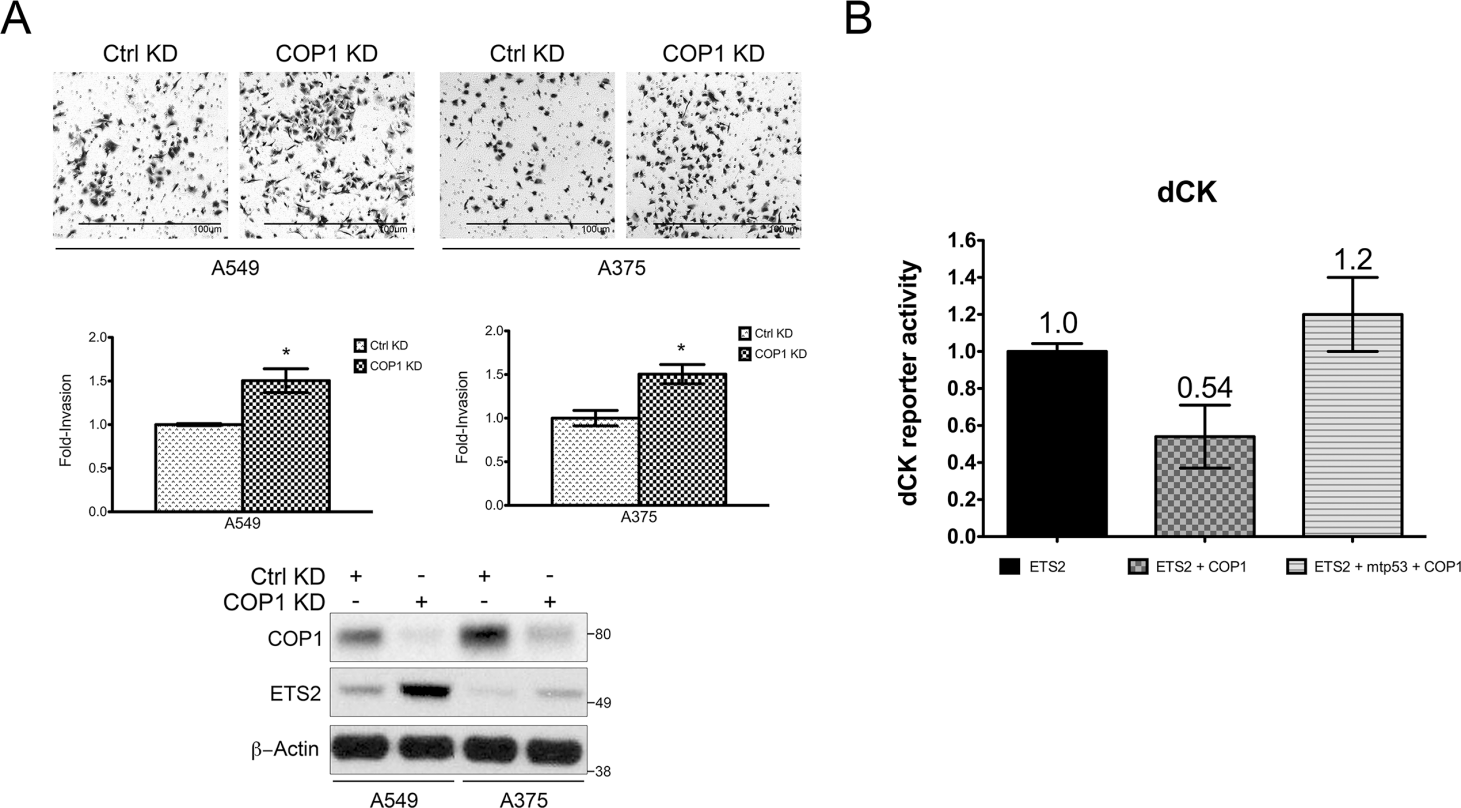
Opposing control of ETS2 by mtp53 and COP1 affects ETS activity (**A**) Hl299 (p53 null) cells were transfected with the indicated plasmids and the dCK promoter luciferase construct. The cells were harvested 24 h later and luciferase activity was measured. (A, B) Shown is the average of two independent experiments with ± SE. (**B**) A549 and A375 (WTp53) cells transfected with either a control or COP1 siRNA were assessed for their invasive activity. Top panel: representative images of invading cells; middle panel: quantitation of invasive cells using a cell counter plug-in for the ImageJ software. Statistical analysis was conducted using an unpaired homoscedastic *t*-test included in the Graph Pad Prism software, *indicates *p* < 0.05 relative to control knockdown; bottom panel: Western blot analysis of siRNA transfected cells.

We previously demonstrated that ETS2 knockdown reduced cell invasion, indicating that ETS2 has a key role in regulating this cellular activity [25]. Since COP1 knockdown induced ETS2, we examined if this also increased cell invasion. In both A549 and A375 cells, COP1 knockdown induced ETS2 protein levels, which correlated with increased invasion (Figure 8A).

In addition to promoting ETS protein degradation, COP1 has been reported to inhibit the ETS protein family's transcriptional activity [15]. Since mtp53 protected ETS2 from COP1/DET1 mediated degradation, we decided to test whether or not it could also prevent COP1 from interfering with ETS2's transcriptional activity. As we had recently reported that ETS2 transcriptionally activates the promoter for deoxcytidine kinase (dCK) [26], we used a dCK promoter luciferase construct for this analysis. In this experiment, we transfected ETS2 with the dCK promoter luciferase construct and examined the impact of co-transfecting COP1. Co-transfection of COP1 resulted in an almost 50% decrease in dCK promoter activity. Strikingly, transfection of ETS2, COP1 and mtp53 fully restored the activity of the promoter construct (Figure 8B). Taken together, these data indicate that mtp53 prevents COP1 from inhibiting ETS2.

## DISCUSSION

We report that COP1 and DET1 are part of an ubiquitin ligase that marks ETS2 for degradation. While this work was in progress, another study demonstrated the same regulation of ETS2 by COP1 [27]. However, despite the overlying similarities in our conclusions, we observed a distinct mechanism by which ETS2 is controlled by this ubiquitin ligase complex. In our study, mutation of the first VP degron site reduced COP1 binding dramatically, whereas mutation of the second VP site did not. We made the surprising observation that DET1 binds to ETS2 independently of COP1, and that mutation of either VP degron site prevents this association. Moreover, we provide the first evidence indicating that DET1 has a role beyond its well accepted scaffolding function in the formation of this ubiquitin ligase complex.

We further characterized the regulation of ETS2 by showing that the COP1/DET1 ubiquitin ligase complex is important for the degradation of ETS2 by CDK10. In addition, we demonstrated that DET1 cannot bind an ETS2 mutant in which the CDK10 phosphorylation sites have been mutated to alanines. This observation suggests that phosphorylation of ETS2 by CDK10 creates a recognition motif for DET1. However, COP1/DET1 are still able to degrade an ETS2 deletion mutant lacking amino acids 205–304 (which harbors the CDK10 sites), it is possible that this portion of the protein is subject to conformational changes that affect ETS2's ability to interact with COP1/DET1. In this case, the phosphorylation sites within this region may play a role in regulating the interaction with COP1/DET1 by impacting its conformation. Nevertheless, the fact that mutation of either the VP sites or CDK10 phosphorylation sites in ETS2 strongly inhibits its interaction with DET1 reinforces the notion that DET1 plays a novel function in the recognition of this ubiquitin ligase substrate. Importantly, we found that mtp53 prevents ETS2 degradation by competing with DET1 for binding to the region harboring the CDK10 phosphorylation sites. We also found that DET1 protein turnover was accelerated in the presence of mtp53. Thus, mtp53 appears to protect ETS2 by both displacing and destabilizing DET1.

Previous studies have proposed a role for COP1 as a tumor suppressor gene [9, 10, 20, 28]. We previously showed that ETS2 knockdown reduces cell invasion and in this study we demonstrated that COP1 knockdown resulted in increased ETS2 levels and increased cell invasion. Taken together, these data identify the control of ETS2 levels as a fundamentally important mechanism for suppressing cancer cell phenotypes. The fact that mtp53 blocks the COP1/DET1 complex from degrading ETS2 and also prevents COP1 from inhibiting the transcriptional activity of ETS2 suggests that mtp53 may exert some of its oncogenic functions by disrupting the homeostatic regulation of ETS2. To our knowledge, it appears that among the different transcription factors that interact with mtp53, only ETS2 is protected from degradation. Given that approximately half of all the mtp53 binding sites in the genome harbor an ETS motif, our data indicate that by protecting its preferred binding partner, ETS2, mtp53 enhances its ability to transcriptionally regulate a multitude of genes involved in cancer progression.

## MATERIALS AND METHODS

### Cell culture, inhibitors, and siRNAs

All cell lines used in this study were purchased from the American Tissue Culture Collection (ATCC, Manassas, VA, USA). A375 cells were cultured in Dulbecco's modified Eagle's medium (DMEM) supplemented with 10% FBS. A549 and PC3 cells were cultured in Roswell Park Memorial Institute medium (RPMI-1640) supplemented with l0% FBS. Cells were seeded in 12-well (0.l25 × 10^6^ cells) and 6-well (0.3 × 10^6^ cells) plastic plates, 60 mm (l × 10^6^ cells) and l45 mm (10 × 10^6^) dishes (Greiner Bio-One, Monroe, NC, USA) and allowed to attach for 24 h prior to the experiment. The Cycloheximide (CHX) and MG-132 inhibitors used in this study were both purchased from Sigma-Aldrich (St. Louis, MO USA). Control, Cdhl, Cul4a, and Skpla targeted siRNAs were purchased from Qiagen (Valencia, CA, USA). RFWD2/COP1 and DET1 targeted siRNA were purchased from Integrated DNA technologies (IDT) (Coralville, IA, USA).

### Plasmid constructs

pCMV-Myc-ETS2 wild-type and deletion mutants, HA-mtp53 R248W, Flag-ETVl, Flag-ETV4, Flag-ETV5, pCMV-β-galactosidase and HA-poly ubiquitin plasmids have been described previously [29]. Human COP1 and DET1 cDNA (GE Dharmacon, Lafayette, CO, USA) was amplified by PCR and subcloned into pCMV-Myc (Clontech, Mountain View, CA, USA). pCMV-Myc COP1 was used as a template to generate the COP1 RING mutant Cl36ACl39A. pCMV-Myc-ETS2 construct was used as a template to generate mutations V52AP53A, Vl87APl88A, and V308AP309A. All mutagenesis was accomplished using the Q5^®^ Site-Directed Mutagenesis Kit from New England BioLabs (Ipswich, MA, USA) and appropriate mutagenesis primers generated by using the NEBaseChanger^™^ tool. Mutations were verified by sequencing.

### Determination of protein stability

A549 cells were transfected with siRNA targeting either Control, Cdhl, COP1, Cul4a, DET1, and Skpla using Lipofectamine^®^ RNAiMAX according to the manufacturer's protocol (Life Technologies, Grand Island, NY, USA). Cell lysates were harvested in 48 h after transfection using RIPA buffer followed by sonication and centrifugation. Estimation of protein estimation was done with the Pierce BCA Protein Assay Kit (Thermo Fisher Scientific, Waltham, MA, USA) prior to subjecting the lysates to Western blotting. Cycloheximide-pulse chase experiments were conducted by replacing the media of A549 cells with media containing CHX and harvesting at different time points. Samples were then processed for Western blotting. A549, H1299, or PC3 cells were co-transfected with and pCMV-β-galactosidase by using Lipofectamine^®^2000 (Life Technologies, Carlsbad, CA, USA) according to the manufacturer's protocol for 24 h. Samples were harvested by adding immunoprecipitation (IP) lysis buffer [4] and rocked for l0 min at 4°C. Lysates were then collected followed by gentle sonication and centrifugation. Samples were normalized to β-galactosidase expression prior to Western blotting by using the Pierce beta-Galactosidase Assay Reagent (Thermo Fisher Scientific, Waltham, MA, USA) according to the manufacturer's protocol.

### Immunoprecipitations

Endogenous interaction of ETS2 and COP1 was detected by treating A549 cells with 20 μM of MG-132 for 6 h. Cells were then harvested by using IP lysis buffer followed estimation of protein concentration. The sample was normalized and split followed by incubation with 1 μg of either monoclonal IgG or ETS2 (E-5) monoclonal antibodies per mg of total protein. The samples were rocked overnight at 4°C, after which they were incubated with Protein G beads (KPL, Gaithersburg, MD, USA) for 2 h at 4°C. Protein G beads were then washed with IP lysis buffer 5×, followed by adding sample loading buffer and boiling for l0 min. Samples were then subjected to Western blotting. ETS2 ubiquitination analysis was done by transfecting A549 cells for 24 h followed by MG-132 treatment 6 h prior harvesting. Cells were harvested by lysing with l% SDS in PBS boiling buffer. Lysates where then boiled at l00°C for l0 min followed by sonication. The samples’ buffer was adjusted to a stringent buffer described previously [30]. Samples were then incubated with 0.5 μg of rabbit ETS2 (C-20) polyclonal antibodies and rocked for 2 h at 4°C. Protein G beads were added to the lysates for l h followed by washing with the stringent buffer 5×. Sample buffer was added to the beads followed by boiling for l0 min. Samples were then subjected to Western blotting.

### Western blotting and antibodies

All Western blotting was conducted as described previously [4]. Primary mouse monoclonal antibodies for Western blots were anti-p53 (DO-l; sc0l26; Santa Cruz Biotechnologies, Dallas, TX, USA), anti-Ets-2 (E-5; sc-365666; Santa Cruz Biotechnologies, Dallas, TX, USA), anti-Myc (9El0; sc-40; Santa Cruz Biotechnologies, Dallas, TX, USA), anti-FLAG (M2; F3l65; Sigma-Aldrich, St. Louis, MO, USA) and anti-HA-probe (F-7; sc-7392; Santa Cruz Biotechnologies, Dallas, TX, USA). Rabbit polyclonal antibodies used for Western blots were anti-Ets-2 (C-20; sc-35l; Santa Cruz Biotechnologies, Dallas, TX, USA) and anti-COP1/RFWD2 (A300–894A; Bethyl, Montgomery, TX, USA). Mouse and Rabbit secondary antibodies were from Cell Signaling Technology (Danvers, MA, USA).

### Luciferase assay

Cell based luciferase assays were performed using the dual luciferase Promega (Madison, WI, USA) kit. H1299 cells were seeded in a white 96-well clear bottom plates. 24 h after transfection, luciferase activity was measured according to manufacturer's instructions. Reporter activity from internal control was used to normalize the test reporter data. Fold changes were calculated by normalizing pGL4-dCK reporter activity towards pGL4 empty vector wells.

## SUPPLEMENTARY MATERIALS FIGURE


