# Supplementary material for: Mutant p53 protects ETS2 from non-canonical COP1/DET1 dependent degradation
Source: Oncotarget. 2016 Feb 9;7(11):12554–67. doi: 10.18632/oncotarget.7275 (PMC4914304; doi:10.18632/oncotarget.7275)
Supplement: Supplementary file 1 [file oncotarget-07-12554-s001.pdf]

Mutant p53 protects ETS2 from non-canonical COP1/DET1 dependent degradation

Supplementary Materials

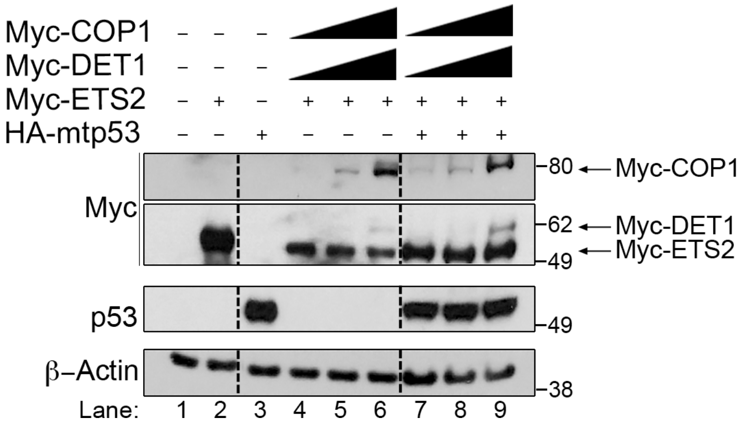

Supplementary Figure S1: Mtp53 protects ETS2 from COP1 and DET11 degradation. Degradation assay of ETS2.H1299 cells were co-transfected with ETS2, COP1 and DET11 with or without mtp53 R248W. Cells were harvested after 24 h, followed by Western blotting. Solid triangle indicates increasing amounts of plasmid.
